# Loss of molars early in life develops behavioral lateralization and impairs hippocampus-dependent recognition memory

**DOI:** 10.1186/1471-2202-15-4

**Published:** 2014-01-04

**Authors:** Masatsuna Kawahata, Yumie Ono, Akinori Ohno, Shoichi Kawamoto, Katsuhiko Kimoto, Minoru Onozuka

**Affiliations:** 1 Department of Prosthodontics & Oral Rehabilitation, Graduate School of Dentistry, Kanagawa Dental University, Yokosuka, Japan; 2 Department of Electronics and Bioinformatics, School of Science and Technology, Meiji University, Kawasaki, Japan; 3 Nittai Jyusei Medical College for Judo Therapeutics, Tokyo, Japan

**Keywords:** Hippocampus, Senescence-accelerated mouse, Cognitive function, Open-field test, Object-recognition test, Behavioral laterality, Dopamine, Chronic stress

## Abstract

**Background:**

Using senescence-accelerated mouse prone 8 (SAMP8), we examined whether reduced mastication from a young age affects hippocampal-dependent cognitive function. We anesthetized male SAMP8 mice at 8 weeks of age and extracted all maxillary molar teeth of half the animals. The other animals were treated similarly, except that molar teeth were not extracted. At 12 and 24 weeks of age, their general behavior and their ability to recognize novel objects were tested using the open-field test (OFT) and the object-recognition test (ORT), respectively.

**Results:**

The body weight of molarless mice was reduced significantly compared to that of molar-intact mice after the extraction and did not recover to the weight of age-matched molar-intact mice throughout the experimental period. At 12 weeks of age, molarless mice showed significantly greater locomotor activity in the OFT than molar-intact mice. However, the ability of molarless mice to discriminate a novel object in the ORT was impaired compared to that of molar-intact mice. The ability of both molarless and molar-intact SAMP8 mice to recognize objects was impaired at 24 weeks of age. These results suggest that molarless SAMP8 mice develop a deficit of cognitive function earlier than molar-intact SAMP8 mice. Interestingly, both at 12 and 24 weeks of age, molarless mice showed a lateralized preference of object location in the encoding session of the ORT, in which two identical objects were presented. Their lateralized preference of object location was positively correlated with the rightward turning-direction preference, which reached statistical significance at 24 weeks of age.

**Conclusions:**

Loss of masticatory function in early life causes malnutrition and chronic stress and impairs the ability to recognize novel objects. Hyperactivation and lateralized rotational behavior are commonly observed with dysfunction of the dopaminergic system, therefore, reduced masticatory function may deplete the mesolimbic and mesocorticolimbic dopaminergic systems to impair the cognitive functions of selective attention and recognition memory in the prefrontal cortex and the hippocampus.

## Background

Masticatory ability has been reported as one of the predictive factors of general health, including cognitive function [1,2]. Impaired masticatory function due to tooth loss causes dietary deficiencies among older adults (reviewed in [3,4]), and the consequent malnutrition status increases the risk of developing cognitive impairment [5,6]. Experimental results in young adult rodents also demonstrate that permanent loss of functional teeth for months impairs digestive and absorptive function by altering the maxillomandibular relationship [7,8] and by reducing secretion of saliva and gastric acid [9,10], even though having teeth extracted does not reduce food consumption [11]. However, the relationship between impaired masticatory function and cognitive deficit for younger individuals remains unclear.

Recently, we showed that sustained interference of masticatory function for 10 days significantly decreased long-term potentiation (LTP) in hippocampal CA1 neurons in young adult rats [12]. Interestingly, these rats also showed increased plasma concentrations of corticosterone and noradrenaline, suggesting that sustained impairment of masticatory function may induce a chronic psychological stress. A recent PET study demonstrated that paralyzing the temporal muscle, a masticatory muscle, for several days results in significant hyperactivation of the hippocampus of young adult rats in a resting state, indicating an increased stress response to uncontrollable mastication [13]. Although our previous study [14,15] reported that a temporal reduction of masticatory function (7 to 10 days) did not alter the cognitive function of young adult subjects, the above evidence raises a hypothesis that long-term interference of masticatory function from a young age causes a complex combination of physical and psychological stress, which may synergistically induce cognitive impairment along with the malnutrition status.

The present study examined the hypothesis that permanent loss of teeth at a younger age affects cognitive function along with growth. We employed the senescence-accelerated mouse prone 8 (SAMP8) as a rodent model for age-related cognitive deficits. SAMP8 mice have a median life span of 13 months and begin to show deficits in learning and memory at 6 months after birth [16]. All upper molar teeth of half of the mice were extracted at 8 weeks of age. At 12 and 24 weeks of age, we performed the object-recognition test (ORT) and the open-field test (OFT) to characterize how long-term reduced mastication modifies the hippocampal-dependent cognitive function of novel object recognition and the general behavior of these mice, respectively. We also conducted follow-up examinations of their body weight through the experimental period to determine whether permanent loss of molar teeth at younger stage of life affects physical growth.

## Results

### Loss of teeth decreases body weight

**Figure 1:**
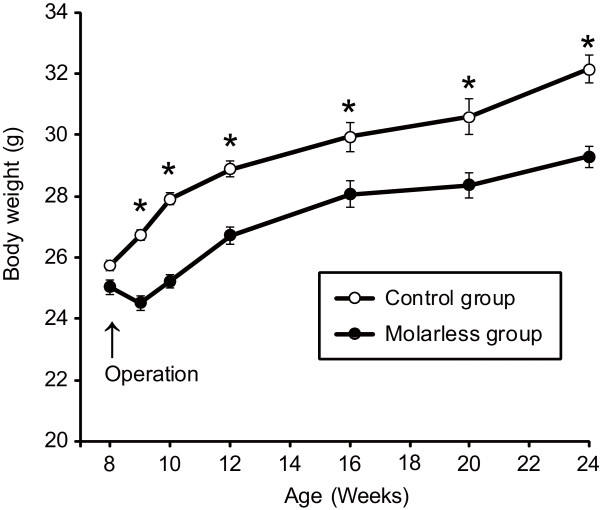
**Change in mean body weight of molar-intact and molarless mice.** Open and filled circles indicate mean body weight of molar-intact (control group) and molarless (molarless group) mice, respectively. We subjected 28 of 58 mice to the behavioral test and sacrificed them at 12 weeks old (15 molar-intact and 13 molarless). Therefore, the number of subjects was 15 each in control and molarless groups at 16, 20, and 24 weeks old. Data are presented as means ± SEM. Asterisks indicate statistically significant differences between groups (9, 10, 12 weeks old Mann–Whitney test, *P* < 0.05 , 16, 20, 24 weeks old Student t-test, *P* < 0.05).

Mean body weight was comparable between the two groups on the day of surgery. The body weight of molar-intact mice increased continuously after the sham operation (Figure 1). On the other hand, the body weight of molarless mice was decreased at 1 week after the extraction, and gradually increased afterwards. Molarless mice showed a significantly smaller mean body weight compared to molar-intact mice over the entire experimental period after the operation.

### Loss of teeth increases locomotor activity without changing the number of entrances to subdivisions of the field

A statistical comparison of total path length in molarless and molar-intact mice in the OFT showed no interaction between age and group, but there were significant main effects of age (2-way ANOVA *F(1,45)* = 9.79, *P* = 0.003) and group (*F(1,45)* = 9.71, *P* = 0.003; Table 1). Further, multiple comparisons showed a significantly increased total path length in molarless mice at 12 weeks of age compared to those in molar-intact mice (*P* = 0.012, Cohen’s d = 0.96). At 24 weeks of age, the total path length was still larger in molarless mice than in molar-intact mice, although the difference did not reach statistical significance (*P* = 0.063). Both molar-intact and molarless mice showed age-related reduction of total path length (*P* = 0.015 and *P =* 0.049, respectively). No significant differences of the number of entrances to subdivisions between groups both at 12 and 24 weeks old were observed.

**Table 1:** The total path length and the number of entrances to the subdivisions in the OFT

	**Total path length (m)**	**Number of entrances (times)**		
		**Center**	**Intermediate**	**Peripheral**
12 weeks				
Control (n = 15)	18.4 ± 0.8	13.4 ± 1	27.1 ± 2	27.8 ± 2
Molarless (n = 13)	20.8 ± 0.5 *	14.0 ± 1	25.2 ± 2	25.9 ± 1
24 weeks				
Control (n = 15)	16.1 ± 0.5 ^†^	7.9 ±1	17.7 ± 2	18.5 ± 2
Molarless (n = 6)	18.3 ± 0.8 ^†^	9.8 ±2	20.5 ± 2	21.7 ± 3

Data are presented as means ± SEM. Asterisks indicate statistically significant differences between groups of the same age (post-hoc Tukey’s multiple comparison, p < 0.05). Daggers indicate statistically significant differences between ages in the same group (post-hoc Tukey’s multiple comparison, p < 0.05).

### Loss of teeth causes early impairment of the ability to recognize objects

Table 2 shows the search time for each object in the encoding and recall sessions of the ORT. At 12 weeks of age, molar-intact mice showed significantly prolonged search time for a novel object than for a familiar object in the retrieval session. However, search time for a novel object of molarless mice was not significantly different from that of a familiar object in the retrieval session. At 24 weeks of age, mice in both groups showed comparable search time for novel and familiar objects. This was expected since SAMP8 mice decline in learning and memory ability from 6 months of age [16]. Interestingly, molarless mice showed an uneven search time for two identical objects in the encoding session. They spent significantly more time with the object placed on the right than with that on the left at both 12 and 24 weeks old (see Methods for the arrangement of objects in the field).

**Table 2:** Search time (seconds) for each object in the encoding and retrieval sessions of the ORT

	**Encoding**		**Retrieval**	
	**Left**	**Right**	**Familiar**	**Novel**
12 weeks				
Control (n = 15)	42 ± 6	43 ± 5	38 ± 5	55 ± 7*
Molarless (n = 13)	44 ± 3	58 ± 5*	41 ± 6	62 ± 8
24 weeks				
Control (n = 15)	46 ± 5	52 ± 7	45 ± 6	45 ± 7
Molarless (n = 6)	33 ± 4	59 ± 8*	46 ± 14	37 ± 9

In the encoding session in which two identical objects were presented, time spent with object placed on the left and on the right (placed at “L” and “R” in Figure 3, respectively) are shown. In the retrieval session, time spent with the familiar object and the novel object are shown regardless of its location. Data are presented as means ± SEM. Asterisks indicate a statistically significant prolonged search time compared to its counterparts (paired t-test, p < 0.05; Cohen’s d > 0.80).

### Loss of teeth develops lateralized behavior

**Figure 2:**
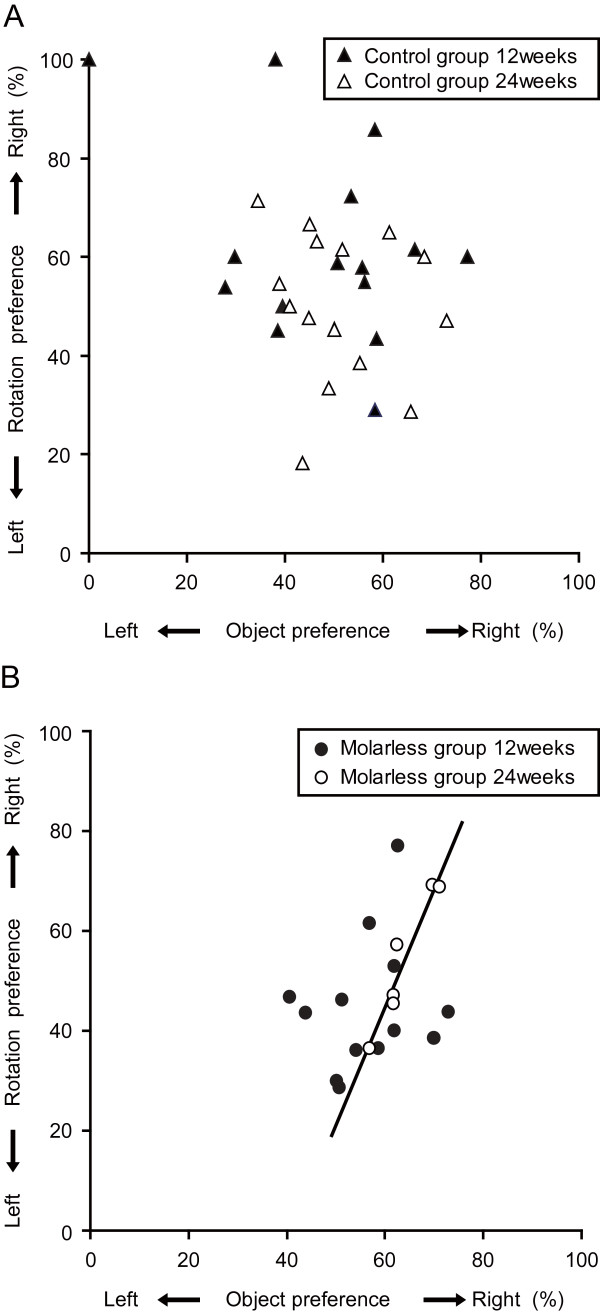
**Scatter plots showing correlation between the side preferences of rotation and object location.** Scatter plots with regression lines for molar-intact mice (control group) are shown in **(A)** and those for molarless mice (molarless group) are shown in **(B)**. Filled triangles: 12 weeks of age (n = 15), open triangles: 24 weeks of age (n = 15), filled circles: 12 weeks of age (n = 13), open circles: 24 weeks of age (n = 6). Rotation preference = 100 × the number of turns to the right / the number of turns in both directions. Object preference = 100 × search time for object placed on the right / search time for both objects.

During the encoding session of the ORT, at which two identical objects were placed in the field, molarless mice did not spend an equal amount of time exploring the two objects, but they spent more time exploring one of the two objects. In contrast, molar-intact mice spent the same amount of time exploring each of the two objects under similar circumstances. Since the mouse was always placed between the two objects at the beginning of the session (See Methods section for detailed arrangement of objects in the test), the preference in object location may originate from a turning-direction preference related to rotational behavior. Therefore, we examined the turning-direction preferences during exploration and its relationship to preference for object location. The directional preferences of rotation and object location were significantly and positively correlated for molarless mice (Pearson's correlation coefficient *r* = 0.963, *P* = 0.002, Cohen’s d = 0.93), but not for molar-intact mice at 24 weeks of age (*r* = −0.152, *P* = 0.589; Figure 2). These results indicate that molarless mice, which prefer to turn toward the right, explored the two objects unequally in the encoding session, spending more time with an object placed on the right side. Even though the correlation was significant for neither molarless mice (*r* = 0.192, *P* = 0.530) nor molar-intact mice (*r* = −0.265, *P* = 0.259) at 12 weeks of age, the correlation coefficient tended to increase with age for molarless mice (Figure 2B), but not for molar-intact mice (Figure 2A).

## Discussion

The key finding of the present study is that sustained loss of masticatory stimulation since an early age not only accelerated the aging process of hippocampal-dependent cognitive function, but also developed abnormal behavior of locomotor hyperactivation and lateralized behavior at their advanced age. Previous studies using the same strain of mice revealed that 7 to 10 days of reduced mastication, caused either by extraction or reduction of molar teeth, impairs spatial learning ability in aged subjects (more than 36 weeks old) with morphological and functional deterioration of hippocampal neurons [14,15,17,18], although the same duration of reduced mastication in young subjects (20–24 weeks at the extraction) did not affect their cognitive function [14,15]. Our results further demonstrated that, even in younger subjects, the sustained reduction of masticatory stimulation for more than two weeks impairs hippocampal-dependent cognitive function in this strain.

Permanent loss of teeth causes loss of somatosensory stimuli from the oral cavity, as well as malnutrition, both of which induce sustained increase of circulating corticosterone concentration [18,19]. Our result in the ORT demonstrated that loss of molar teeth for a long period of time may be a chronic stress that accelerates age-related cognitive impairment. The sustained hyperactivation of the hippocampal neurons via activation of stress-induced glucocorticoid receptors (GRs) causes the functional and morphological decline of these neurons [20]. Attenuated neuronal responsiveness to new stimuli in the hippocampus impairs the spatial-learning ability, possibly interfering with the induction of LTP in hippocampal neurons [21]. Tsutsui et al. [22] reported that the sustained reduction of masticatory stimulation induced loss of pyramidal cells in the hippocampal CA1 and CA3 regions. As CA1 and CA3 regions are critical to recognizing familiar item or familiar spatial information [23] and to detecting a novel visual object in a spatial-temporal context [24], respectively, permanent loss of teeth may impair hippocampal cognitive functions of both spatial and episodic memory.

Attenuated hippocampal function may further cause over-secretion of corticosteroids as the hippocampus is a target brain region of corticosterone regulating its negative-feedback system [25]. Nyakas et al. [20] reported that activation of GR is essential for the expression of exploratory activity in a novel environment, and that animals with partial lesions in their hippocampi engage in enhanced exploratory activity with increased uptake of glucocorticoids in the remaining neurons in the hippocampus. The locomotor hyperactivation observed in molarless mice in the OFT suggests that chronic stress impairs the function of hippocampal neurons, resulting in hyperactivation of GRs.

Chronic stress derived from reduced mastication has also been reported to enhance oxidative stress [26] and reduce the response of the dopamine (DA) neurons in the hippocampus, impairing the learning ability in the step-through passive avoidance test [27]. The DA projection from the midbrain dopamine cells of the ventral tegmental area (VTA) to the hippocampal neurons mainly regulates the late phase of LTP, which is essential for the settlement of long-term memory [28]. Although we did not measure the hippocampal LTP in this study, accumulating results suggest that the chronic stress caused by reduced mastication has at least two possible neuronal pathways to impair the hippocampal-dependent learning ability: (1) increased corticosterone directly interferes with the induction of hippocampal LTP, and (2) reduced DA responsiveness in the hippocampus prevents the settlement of the LTP.

Lateralized behavior of molarless mice observed in the ORT also suggests the possible involvement of an attenuated DA system in the hippocampus. The prefrontal cortex (PFC) is another projection site of DA neurons from the VTA, and chronic stress causes a hypodopaminergic state in the PFC [29], as well as in the hippocampus [30] with an impaired spatial working memory [31]. Interestingly, exposure to an uncontrollable stressor causes a lateralized alteration of DA consumption in the PFC [32], which closely relates to lateralized rotational preferences [33]. The age-dependent development of the lateralized preference of rotational behavior in molarless mice may indicate the selective and progressive reduction of neuronal transmission of the DA system.

Another possible hypothesis supporting the attenuated DA system in the molarless mice is iron deprivation caused by extraction-induced reduction of gastric acid secretion [10]. The iron is a key element in DA synthesis, and DA deficit in the brain is generally recognized as a pivotal for hyperactivity and pathologic lateralization of cognitive functioning that are commonly observed in attention-deficit/hyperactivity disorder (ADHD) [34]. In fact, an excessive spontaneous motor activity and a deficit in selective attention are key signatures of the rodent model of ADHD [35], which corresponds to the behavior of molarless mice at 24 weeks of age.

A limitation of this study is that it is difficult to separate the influence of malnutrition from psychological stress on the development of cognitive and behavioral deficit in the molarless mice. Considering that either food deprivation with intact masticatory function or molar extraction with powder-diet feeding causes impairment of the hippocampus-dependent memory function, as well as attenuation of the DA system [36,37], these two factors might have an additive effect. Further examination of the serum ferritin levels or administration of DA agonist/antagonist in the current rodent model is needed to elucidate the effect of masticatory function on the systemic development in detail. Uneven dimensions of subdivisions defined in the OFT might dismiss the preference for peripheral and/or intermediate areas, although it would not affect the interpretation of the result of comparable number of entrances to the subdivisions between molarless and control groups.

## Conclusions

Sustained reduction of masticatory stimulation impaired hippocampal-dependent learning ability even in young animals, which was associated with increased locomotor activity in exploring a novel environment and imbalanced rotational preference during exploring behavior. These results suggest that reduced mastication at an early stage of life may be a chronic stress that accelerates the aging process of the hippocampus.

## Methods

### Ethics Statement

This study was reviewed and approved by the Animal Care and Use Committee of Kanagawa Dental University and conformed to the Guidelines for Care and Use of Laboratory Animals of the National Institutes of Health.

### Animals

We used 60 male SAMP8 mice (Nihon SLC Co. Ltd., Shizuoka, Japan), aged 7 weeks when they were purchased. The mice were housed in groups of 8 in plastic cages under temperature- and humidity-controlled conditions (22 ± 3°C, 55 ± 2%) with free access to pelleted food and water. We used standard diet without specific dietary supplement. Since the maxillomandibular incisors and mandibular molar teeth were kept intact even in the molar-extracted (molarless) mice, all mice could eat pelleted food throughout the experiment. The light–dark cycle was set at 12 h (lights on from 7:00 AM to 7:00 PM). Since two mice died during the manipulation of molar extraction, we had 30 and 28 mice for control (molar intact) and molarless groups, respectively. Among them, 15 and 13 of control and molarless mice, respectively, were sacrificed at 12 weeks of age after the behavioral tests described below. The rest of 30 mice (15 for each group) were kept until 24 weeks of age and subjected to the behavioral tests. Due to the technical error of the video-capture system, behavioral data from eight 24 weeks-old molarless mice were not available.

### Removal of molar teeth

At 8 weeks of age (from day 56 to 62), all mice were deeply anesthetized with sodium pentobarbital (10 mg/kg *i.p.*; Somnopentyl, 10 ml/kg, Kyoetsu Seiyaku Co. Ltd., Tokyo, Japan). Our preliminary experiment demonstrated that a root of molar tooth began to bend in the alveolar bone from 8 weeks of age. We therefore performed extraction at this age to avoid the possibility to leave stumps of broken tooth upon extraction which may cause an additional stress from pain. Anesthetized mice were lightly restrained in a supine position on a mesh grid and their mouth was kept open using cotton string tied to their maxillomandibular incisors. Half of the mice had all upper (maxillary) molar teeth extracted using dental tweezers (molarless group; [17]). The time required for extraction was no longer than 10 min. The other half had the same manipulation except that the molar teeth were not extracted (control group). After the manipulation the mice were recovered under a heat lamp for 10 min and then returned to the home cage. The mice were housed separately with respect to the molarless or molar-intact mice in a group of 4–6 per cage during the post-operative period. We did not administer any post-operative antibiotics and other medicaments, since our preliminary experiment demonstrated that this molar extraction procedure does not cause any periodontal inflammation. All mice were weighed prior to the operation and at 9, 10, 12, 16, 20, and 24 weeks old.

### Open-field test (OFT)

**Figure 3:**
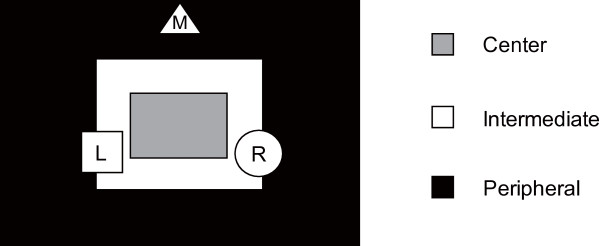
**An overhead view of the open field used in the OFT and ORT.** In the OFT, we divided the oblong field (55 cm × 40 cm) into three subdivisions of center (gray; 15 cm × 10 cm), intermediate (white; 25 cm × 20 cm), and peripheral (black) and counted the number of entrances for each subdivision in addition to measuring the total path length. In the ORT, two objects were placed at the positions indicated as “R (object on the right)” and “L (object on the left)”, and time spent to explore each object was measured. In all experiments, the mouse was always placed at the position “M” at the beginning of the test, with its face oriented to the wall.

The OFT was carried out to investigate spontaneous motor activity of mice in a novel environment. The open field consisted of an oblong arena (55 × 40 cm) surrounded by walls of 40 cm height, as described previously [38], arranged in a sound-isolated room. A video camera (DCR-HC1000, Sony Inc., Japan) was suspended from the ceiling above the arena to observe and record the behavior of animals. The field was constantly illuminated with a dim light (12 Lx at the floor of the arena). The animals in their home cage were brought to the experimental room at least 1 h before the beginning of the first trial of the day. At the beginning of each trial, the mouse was placed at the middle of the long side of the arena, closely facing the wall. The field was divided into three oblong subdivisions of center (15 cm × 10 cm), intermediate (25 cm × 20 cm), and peripheral (55 cm × 40 cm) areas for scoring ambulatory activity (Figure 3). The arena was first halved into two subdivisions of intermediate and peripheral areas, and the center subdivision was further added to discriminate the behavior of crossing through the center part of the arena from that of travelling around the outer boundary of the intermediate area. The arena was isolated with a light-blocking curtain and the home cage was covered with a cloth during the whole experiment so that the mice in the home cage could not see the testing apparatus. Since it was also a part of the ORT procedure to acclimatize the mice to the field in which novel objects are placed, all mice were allowed to freely explore the field for 15 min once a day for 3 consecutive days preceding the ORT. We measured total path length and the count of entrances into each subdivision using a video tracking system (Top Scan, Clever Sys Inc., VA, USA) for the first 5 min on the first experimental day as parameters of OFT. Total path length was defined as a cumulated travel distance of the center of gravity of the mouse body in the arena for the 5 min period. Entrance to a subdivision was counted if the center of gravity of the mouse body passed through the border of the subdivision from outside to inside. After the completion of each trial, the animal was returned to the home cage and the field was cleaned with 30% ethanol. All experimental procedure took place between 6–8 pm.

### Object-recognition test (ORT)

The ORT is a method to study the hippocampal-dependent long-term recognition memory in mice using their tendency to interact more with a novel object than with a familiar one. The protocol of ORT was adapted from Ennaceur and Delacour [39] as previously described [40]. The test objects were a glass and a ceramic cup, which were different in shape, texture and color. We tested the mice on the following day after completing the OFT. In the first (encoding) session, two identical objects were placed in the same field as used in the OFT (Figure 3) and the animal was allowed to freely explore for 5 min. It was considered that the animal was exploring the object when (1) the head of the subject was oriented toward the object within 2 cm of the object (watching, touching or sniffing the object) or (2) the center-of-gravity of the subject’s body was located inside the area where the object was placed (standing along the surface or climbing up on the object). The animals in their home cage were brought to the experimental room at least 1 h before the beginning of the first trial of the day as in case of the OFT. The first session was carried out between 6-7 pm. After the completion of each trial, the animal was returned to the home cage and the field was cleaned with 30% ethanol. One hour later the second (retrieval) session took place, in which one of the objects was replaced by a different one, and exploration was again scored for 5 min. We chose the novel object alternately and the position to place the novel object was counterbalanced among subjects. Results were expressed as duration of time spent with each object (defined as search time).

### Laterality preferences during object exploration

**Figure 4:**
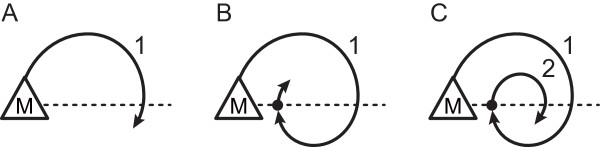
**Definition of turns during object exploration.** A turn, either to the left (counterclockwise traveling) or to the right (clockwise traveling), was defined if the mouse turned more than 180° from the original position indicated as “M” **(A)**. If the mouse consecutively turned more than 360° in one direction at once, we counted the first 360° as one turn and then started counting the new one. For example, a consecutive turn from 180° to 540° was counted as single turn **(B)** and that from 540° to 720° was counted as two turns **(C)**.

The center-of-gravity position of a mouse as a function of time was obtained from the video tracking system. A 5-min recording of position in the encoding session of ORT was divided into ten 30s periods and displayed separately on the computer screen to facilitate scoring. A turn, either to the left (counterclockwise traveling) or to the right (clockwise traveling), was defined if the mouse turned more than 180° (Figure 4A). If the mouse consecutively turned more than 360° in one direction at once, we counted the first 360° as one turn and then started counting the new one (Figure 4B and C). For each animal, the counting of any successive number of turns in a particular direction was interrupted when the animal presented a shift in its initial direction, or even when it explored an object. The side preferences of rotation (rotation preference) were determined as a percentage of the number of turns to the right to that of total turns. We further defined the preference of object location (object preference) as a percentage of the time spent at the object on the right to total time spent for exploration of both objects. The correlation between the rotation preference and object preference was investigated.

### Statistics

All values shown are mean ± SEM. All data were subjected to a Shapiro-Wilk Test of Normality. A paired t-test or unpaired t-test was used when the data was normally distributed, otherwise a nonparametric Mann–Whitney U test was used. We compared total path length and the count of entrances into each subdivision in OFT using the two-way analysis of variance (ANOVA) test and the post-hoc Tukey’s multiple comparison. We used the Pearson product–moment correlation coefficient to determine the significance of correlation between the preferences of rotation and that of object location. We consider *P* values <0.05 to be statistically significant.

## Competing interests

The authors declare that they have no competing interests.

## Authors̱ contributions

MK, AO, and SK carried out behavior studies, performed the statistical analysis, and drafted manuscript. YO, KK and MO conceived of the study, participated in its design and coordination and helped to draft the manuscript. All authors read and approved the final manuscript.
